# Retrocaval Ureter: Report of Two Cases

**DOI:** 10.1155/2019/2815748

**Published:** 2019-11-03

**Authors:** Henry Atawurah, Patrick Opoku Manu Maison, Mohammed Owusu-Ansah, Alvin Asante-Asamani

**Affiliations:** ^1^Department of Surgery, School of Medical Sciences, College of Health and Allied Sciences, University of Cape Coast, Cape Coast, Ghana; ^2^Department of Radiology, Cape Coast Teaching Hospital, Cape Coast, Ghana; ^3^Department of Surgery, Cape Coast Teaching Hospital, Cape Coast, Ghana

## Abstract

Retrocaval ureter (RCU) is a rare congenital anomaly in which the ureter passes posterior to the inferior vena cava (IVC). A little over 200 cases have been reported worldwide since Hochstetter's first report in 1893. We present two cases of retrocaval ureter which were successfully managed at the Cape Coast Teaching Hospital in Ghana. *Case 1.* A 55-year-old woman presented with a history of dull right flank pain of 2 years duration. Physical examination and basic laboratory investigations performed on her were normal. Abdominal ultrasound showed right hydronephrosis and a retrograde right ureteropyelogram (RPG) showed right hydroureteronephrosis with an “S” shaped proximal ureter. A diagnosis of retrocaval ureter was made and confirmed at surgery. *Case 2.* A 25-year-old man presented with dull intermittent right flank pain of 1 year duration. Clinical examination and laboratory investigation were normal. Abdominal ultrasound showed right hydronephrosis and a CT urogram made a diagnosis of retrocaval ureter which was confirmed at surgery. *Conclusion.* Retrocaval ureter is a rare congenital anomaly that is now increasingly being reported. Surgical treatment of symptomatic cases successfully relieves symptoms.

## 1. Introduction

Retrocaval ureter (RCU) is a rare congenital anomaly in which the ureter passes posterior to the inferior vena cava (IVC) [1]. This anomaly occurs between the 4^th^ and 8^th^ weeks of intrauterine development and is due to abnormal formation of infrarenal IVC from anteriorly located subcardinal vein instead of supracardinal vein which are located posteriorly [2]. In normal circumstances, the infrarenal IVC originates from dorsally located supracardinal vein, but when it develops from ventrally located subcardinal vein, the ureter is trapped posteriorly leading to pre-ureteral vena cava [2].

This rare anomaly, first described in 1893 by Hochstetter in a cadaver [3] and the first clinical diagnosis in 1940 by Harrill [4], has an incidence of 0.06–0.17% worldwide [5]. Very few cases have been reported from Sub-Saharan Africa including four from Nigeria and two from Ghana [6, 7]. Though it no longer attracts the curiosity witnessed in the 1940s, it is still worth reporting on it especially in the case of successful surgical correction.

We present two cases of retrocaval ureter which were successfully managed at the Cape Coast Teaching Hospital in Ghana. This is the second case report from Ghana.

### 1.1. Case 1

**Figure 1:**
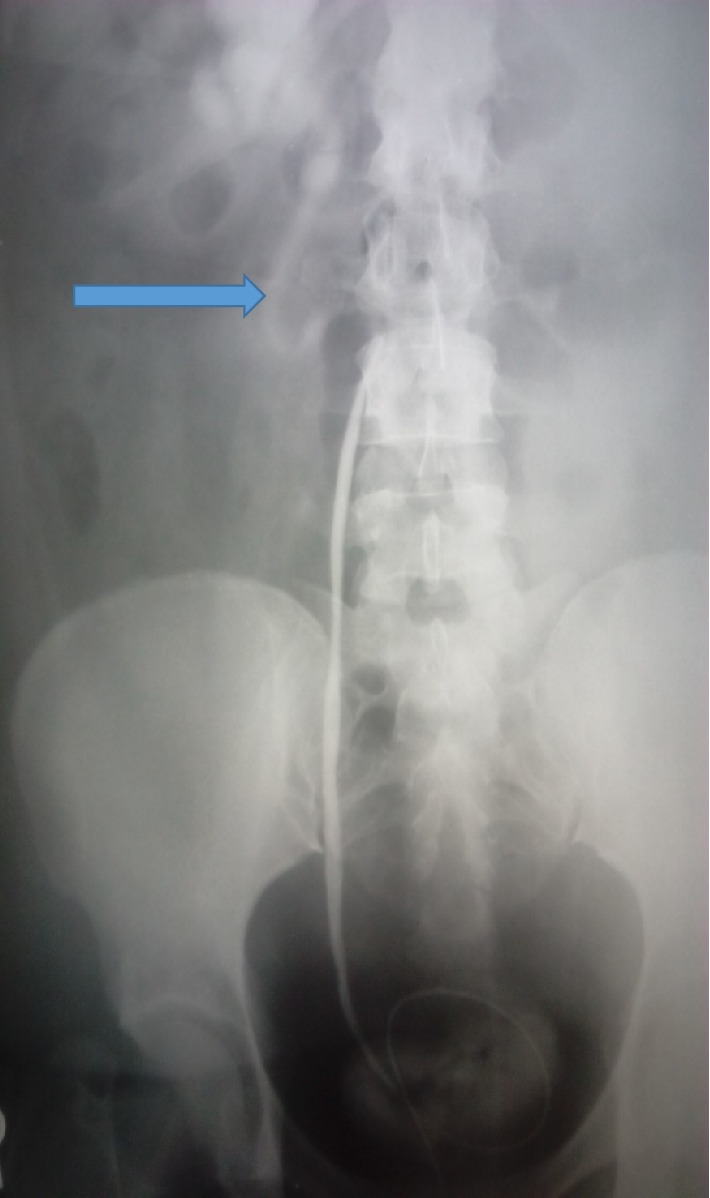
Retrograde ureteropyelogram showing an “S” shaped or “fish hook” deformity of the proximal ureter (Bold arrow).

A 55-year-old woman presented with a history of dull right flank pain of 2 years duration. She was otherwise well and clinical examination of the abdomen was normal. Laboratory evaluation including urinalysis, full blood count, urea, creatinine and electrolytes were within normal limits. Abdominal ultrasound showed right hydronephrosis and a retrograde right ureteropyelogram (RPG) (Figure 1) showed right hydroureteronephrosis with an “S” shaped or “fish hook” deformity of the proximal ureter, which terminated abruptly. A diagnosis of retrocaval ureter was made and the findings at operation were that of right retrocaval ureter, proximal dilated ureteral segment and a normal distal segment lying between the aorta and IVC. The redundant retrocaval segment was mobilized and excised, and end-to-end anastomosis was achieved over a JJ stent. The patient's symptoms resolved at follow-up.

### 1.2. Case 2

**Figure 2:**
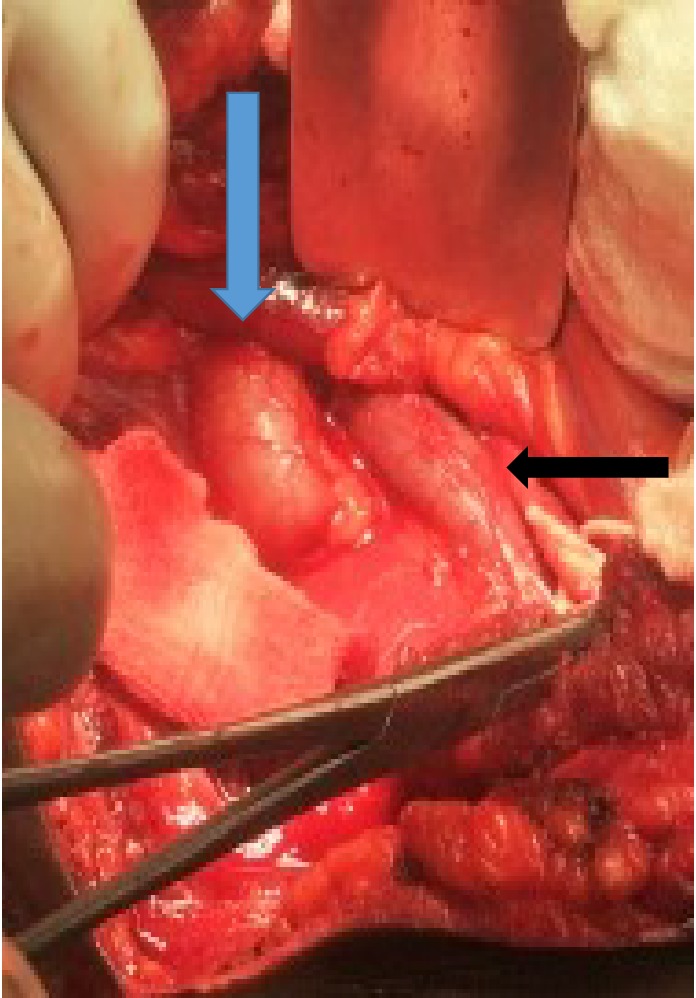
Operative photograph showing the right proximal ureter (blue arrow) coursing behind the inferior vena cava (black arrow).

**Figure 3:**
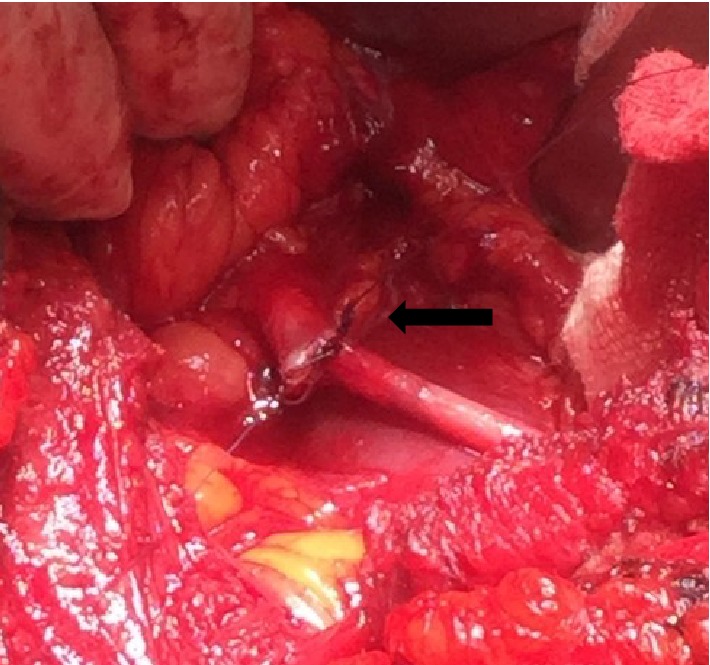
Excision with end to end ureteral anastomosis done over a JJ stent (arrow).

A 25-year-old man presented with a history of dull intermittent right flank pain of 1 year duration. He had no history of fever, dysuria, haematuria or weight loss. Clinical examination of the abdomen was within normal limits. Laboratory evaluation was normal. Abdominal ultrasound showed right hydronephrosis with proximally dilated ureter. A CT urogram to delineate the ureter made a diagnosis of retrocaval ureter with proximally dilated ureter which was confirmed at surgery (Figure 2). Excision of the retrocaval segment with end to end ureteral anastomosis over a stent was done (Figure 3). The patient's symptoms resolved at follow-up.

## 2. Discussion

Retrocaval ureter is a rare congenital anomaly that is caused by an abnormal formation of infrarenal IVC from anteriorly located subcardinal vein instead of supracardinal vein which are located posteriorly [2]. It entraps a segment of the proximal ureter, resulting in the ureter wrapping around the IVC. Therefore, it is also known as circumcaval ureter or preureteral vena cava [8]. This anomaly is rare with an incidence of 0.06–0.17% worldwide [5]. A little over 200 cases have been reported worldwide since Hochstetter's first report in 1893 [3]. There are very few reported cases from Sub-Saharan Africa but Ahmed et al. who managed four patients between 2010 and 2017 in Nigeria believe that the condition is probably under-reported [6]. In Ghana, Kyei et al. reported on two patients with retrocaval ureter in 2011 [7].

It is three times more common in males than in females [9, 10]. It usually occurs on the right side but can be on the left side in patients with situs inversus, duplication of IVC or persistent left subcardinal vein [11, 12]. Although congenital, it usually becomes symptomatic in the third or fourth decade of life due to hydronephrosis from compression of the ureteral segment by the IVC against the psoas muscle, ureteral kinking or from an adynamic retrocaval ureteral segment [2, 13]. The symptoms include flank or abdominal pain and haematuria. Urinary infection, stone formation, and renal dysfunction may complicate the ureteral obstruction. Some patients may present with symptoms earlier than the third or fourth decade of life and it may also be asymptomatic; discovered only during imaging or surgery for unrelated conditions or at autopsy [14]. In this series, one patient was a male in the third decade and presented with right flank pains. However, the other patient was a female in her sixth decade. Retrocaval ureter may be associated with other anomalies mainly in the urogenital and cardiovascular systems. Some of the associated anomalies include duplication of IVC, situs inversus, imperforate anus, oesophageal atresia, myelomeningocele, renal agenesis, horse shoe kidney, ureteral duplication, congenital absence of vas deference, hypospadias, intestinal malrotation, VACTERL and Turner's branchial arch [15].

There are two types of RCU. The more common form, Type I, is a low-loop of the proximal ureter. The obstruction is typically at the edge of the iliopsoas muscle, at which point the ureter deviates cephalad before passing behind the vena cava. This results in a proximal ureteral dilation and hydronephrosis, demonstrating a fishhook, reverse-J, or S-shaped ureteral curve as observed on the retrograde ureteropyelogram of the first patient in this series. The less common Type II is a high-loop of the ureteropelvic junction. The proximal ureter passes behind the vena cava at a higher level, with the renal pelvis and upper ureter lying almost horizontal before encircling the vena cava [1]. It has a lesser degree of hydronephrosis or none and demonstrates a sickle shaped smooth curve on IVU [1]. The two patients in this series had type I RCU.

RCU is usually diagnosed with an intravenous urogram (IVU), retrograde pyeloureterogram (RGP) or computerized tomography (CT) scan [16]. Spiral CT scan is considered the investigation of choice compared to IVU because it can outline both the ureter and IVC [17]. Magnetic Resonance Imaging (MRI) may be preferable to CT scan as it can delineate the course of the ureter and IVC with no exposure to radiation as compared to IVU or CT scan [5]. Nuclear renal scan is useful to evaluate degree of the obstruction and renal function [18].

Repair usually involves open or laparoscopic resection of the redundant retrocaval ureteral segment, anteposition, and ureteroureteral or ureteropelvic anastomosis [19].

## 3. Conclusion

Retrocaval ureter is a rare congenital anomaly that is now increasingly being reported. The low clinical incidence may be due to a number of asymptomatic cases that are not diagnosed in the patient's lifetime. Surgical treatment of symptomatic cases successfully relieves symptoms.
